# Transient Response of Miniature Piezoresistive Pressure Sensor Dedicated to Blast Wave Monitoring

**DOI:** 10.3390/s22249571

**Published:** 2022-12-07

**Authors:** Kevin Sanchez, Bilel Achour, Anthony Coustou, Aurélie Lecestre, Samuel Charlot, Maylis Lavayssière, Alexandre Lefrançois, Hervé Aubert, Patrick Pons

**Affiliations:** 1 Laboratoire d’Analyse et d’Architecture des Systèmes (LAAS-CNRS), Centre National de la Recherche Scientifique (CNRS), Institut National Polytechnique de Toulouse (INPT), Université de Toulouse, 7 Avenue du Colonel Roche, 31031 Toulouse, France; 2 Commissariat à l’Energie Atomique et aux Energies Alternatives (CEA), Direction des Applications Militaires-(DAM), 46500 Gramat, France

**Keywords:** blast wave, transient response, piezoresistive sensor

## Abstract

Blast waves generated by energetic materials involve very fast time variations in the pressure. One important issue for blast wave metrology is the accurate measurement (typical precision in the range of ±5% or better) of the static overpressure peak. For most near field configurations, this measurement requires ultra-fast sensors with response times lower than a few microseconds. In this paper, we design, model, fabricate and characterize a new ultra-fast sensor using piezo-resistive gauges at the center of a miniaturized and rectangular silicon membrane. When a pressure step of 10 bar is applied to the membrane, the signal delivered to the sensor output presents dampened oscillations, with a resonant frequency of 20.6 MHz and quality factor of 24,700 ns after the arrival of the shock wave. After removing undesirable drifts that appear after 700 ns, we may expect the sensor to have a response time (at ±5%) of 1.2 µs. Consequently, the proposed pressure sensor could be advantageously used for the accurate measurement of static overpressure peaks in blast wave experiments.

## 1. Introduction

**Figure 1:**
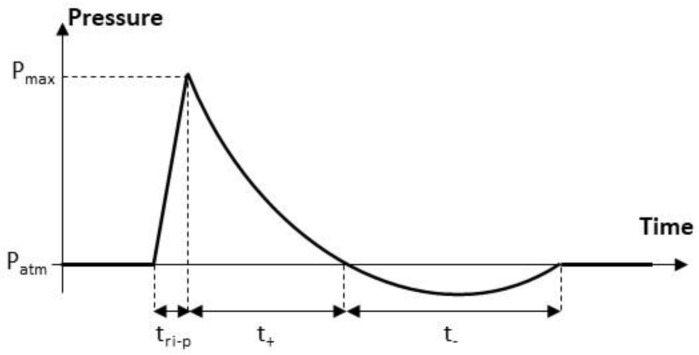
Typical pressure variation during an air blast experiment ($P_{atm}$ denotes the atmospheric pressure before the shock wave, $P_{max}$ is the pressure peak, $t_{ri-p}$ is the rise time of the pressure; $t_{+}$ and $t_{-}$ denote the durations of the positive and negative phases, respectively).

The detonation products of an energetic material create a blast wave and then very fast time variations in the pressure (see Figure 1) [1,2]. These variations are composed of the following three successive phases: (1) the abrupt increase of duration $t_{ri-p}$ (<10 ns) from the atmospheric pressure $P_{atm}$ to the maximum pressure $P_{max}$, (2) the positive phase of duration $t_{+}$, where the pressure decreases exponentially from the peak value $P_{max}$ to the atmospheric pressure $P_{atm}$ value ($t_{+}$ ranges typically from 100 µs or less to 1 ms or more, depending on the mass of the explosive charge and the charge-to-sensor separation distance); and (3) the negative phase of duration $t_{-}$, where the pressure takes its minimum value and returns to the atmospheric pressure value ($t_{-}$ depends on the mass of the explosive charge and the charge-to-sensor separation distance, and is greater than the duration $t_{+}$ of the positive phase).

Blast wave metrology consists mainly of characterizing the pressure variation over the three phases and is widely used for, e.g., the development of blast-resistant civil architecture [3] or the creation of explosive charges [4]. A key descriptor of a blast wave is the overpressure $P_{r-max}$ ($P_{r-max}$ *=* $P_{max}$ *−* $P_{atm}$) generated by an explosion. Its accurate estimation may be used to validate state equations for detonation products and for hydrodynamic and thermo-chemical numerical codes, to characterize the performance of explosive charges and blast–structure interaction and to ensure the pyrotechnic safety from the specification of effective danger zones. For all these applications, the minimum accuracy required for the measurement of the maximum overpressure $P_{r-max}$ is typically 5% in order to obtain sufficiently reliable data. However, the aim is to eventually reach an accuracy of 1%, as is already the case for the metrology of other blast wave parameters, such as the shock-wave velocity obtained by chronometry. Therefore, the accurate and direct determination of the overpressure requires the use of sensors with a response time that is much shorter than the duration $t_{+}$ of the positive phase.

The sensors that are currently available on the market, which specifically address blast wave monitoring, are based on the use of a disc made of piezoelectric material (Table 1). The stress applied to this disc generates charges (by the piezoelectric effect), which are collected by electrodes and processed by a nearby electronic circuit.

The piezoelectric materials used to manufacture these sensors are quartz crystals, tourmaline crystals and polyvinylidene fluoride polymer films.

Quartz crystals have a typical thickness of a few hundred microns and require compression mounting to increase their mechanical resonant frequency, meaning they are more sensitive to packaging conditions [5,6]. However, quartz has the advantage of a low temperature dependence in some crystallographic configurations. This material is, therefore, preferred for the manufacture of sensors in high-temperature conditions. Due to the large mass of the quartz disks, they are also sensitive to accelerations generated by mechanical vibrations. For this reason quartz disks that are not subjected to pressure are used in these sensors to compensate for acceleration. The resonant frequency of these sensors ranges from 200 kHz to 500 kHz. These sensors are both available in face-on (PCB-113B, KISTLER-601C/603C) and side-on (PCB-137B, KISTLER-6233A) configurations.

Tourmaline crystals do not require (unlike quartz) compression mounting. However, this material also has pyroelectric properties [7] and a maximum operating temperature of 50 °C is recommended in the datasheet of the manufacturer. This sensor (PCB-134A) is recommended for shock-wave tube measurements and has a resonant frequency of 1500 kHz.

Polyvinylidene fluoride (PVDF) polymer films (few tens of microns thick) have higher resonant frequencies (4 MHz) than quartz or tourmaline crystal. However, their piezoelectric properties degrade with temperature [8] and a maximum operating temperature of 65 °C is recommended in the datasheet of the manufacturer. These sensors (M60 and M100) are suitable for shock-wave tube experiments when measurements at high frequency are required, but they suffer from low cut-off frequency.

For most of these commercial sensors, the rise time is low (<1 µs) in the so-called face-on configuration, that is, when the blast wave direction is normal to the surface of the sensor. However, in this configuration, the diameter of the sensitive element (rarely communicated by the manufacturer) is not critical. The rise time is much higher (>3 µs) in the so-called side-on configuration, i.e., when the direction of the blast wave is tangential to the surface of the sensor. This is due to the time required by the wave to travel along the surface diameter. Experiments carried out in recent years at CEA-Gramat, for side-on configurations and near field conditions, have shown that the best commercial sensors do not allow the direct measurement of $P_{r-max}$ within ±10%.

Most of the studies are focused on sensors with optical transduction that minimize the disturbance caused by electromagnetic interference. The following two types of concepts are used: (1) the distributed Bragg reflector based on the modification of the refractive index with strain. These devices are dedicated to the measurement of very high pressures (>1 kbar), which generally occur during the impact of projectiles on targets [9,10] (they will not be detailed here, because this is not the pressure range targeted in our work); (2) the Fabry-Perot cavity modulated most often by the deflection of a membrane [11,12,13,14,15,16,17,18] and more rarely by the crushing of a low Young’s modulus layer) [19]. These sensors are more suitable for low to moderate pressures (typically, from 100 mbar to 100 bar).

**Figure 2:**
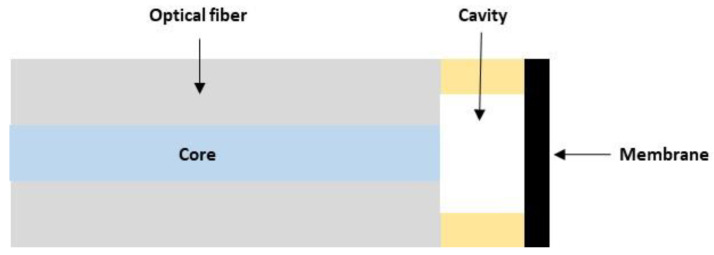
Topology of Fabry–Perot cavity sensor with a membrane as a mechanical transducer.

Fabry–Perot cavity sensors use an optical fiber with an external diameter of 125 µm. The interrogation zone is composed of the core of the 10 µm diameter fiber. Membrane sensors require the construction of a cavity that separates the end of the fiber core from the surface of the membrane (Figure 2). Early work in the 2000s [11,12,13] used a metal membrane cut from a 3 µm thick copper foil and bonded to a zirconium ferrule. The fiber was then bonded to the inside of the ferrule. Given the technological limitations (control of membrane dimensions and cavity size), the researchers then turned to the use of microtechnologies for the fabrication of cavities and membranes [14,15,16]. The membrane made of silicon oxide or silicon nitride, with a thickness of 1 µm, was produced on a silicon substrate. Chemical or deep reactive ionic etching (DRIE) machining of the silicon allows the fabrication of the cavity and access to the fiber. Microtechnologies have allowed better dimensional control of the membrane and cavity dimensions, but the fiber is still transferred and bonded to the silicon substrate, which is not reliable due to the properties of the adhesive. Since 2012 [17,18], the cavity has been made directly at the optical fiber either by localized etching or by fusion welding of a ring. A 3 µm thick silicon oxide membrane is then directly transferred to the fiber and welded by fusion.

These optical sensors have a rise time of 0.2 µs in the face-on configuration. This rise time is close to the one achieved by the best commercial sensors based on piezoelectric disks, but the resonant frequency is about ten times higher. Another advantage of these optical sensors is the small diameter (between 50 µm and 100 µm) of the pressure-sensitive part (deformable membrane), which guarantees both the short travel time of the shock wave on the sensitive part in the side-on configuration, and a very good spatial resolution of the pressure measurement. Given the low mass of the membrane, these sensors are also not very sensitive to acceleration and do not require compensation [5]. However, optical sensors suffer from the following two critical weaknesses: they do not allow collective manufacturing and are not suitable for multi-sensor integration.

To overcome the aforementioned issues of the available pressure sensors for blast wave monitoring, we report in this paper the design, modelling, fabrication and characterization of a new ultra-fast sensor, using piezo-resistive gauges at the center of a miniaturized and rectangular silicon membrane [20,21,22,23]. The sensor is dedicated to the measurement of pressure during blast experiments and for overpressure peaks that range from 1 bar to 70 bar. The paper is organized as follows. Section 2 reports the technical specifications of pressure sensors for the accurate measurement of overpressure peaks during blast wave experiments. Section 3 presents the experimental set-up used for the dynamic characterization of the sensors in a shock-wave tube. All constitutive parts of the set-up are detailed in Section 4, Section 5 and Section 6. Section 7 discusses the obtained measurement results and, the conclusions and perspectives of this work are finally drawn in Section 8.

## 2. Technical Specifications of Pressure Sensor for the Accurate Measurement of $P_{r-max}$

**Figure 3:**
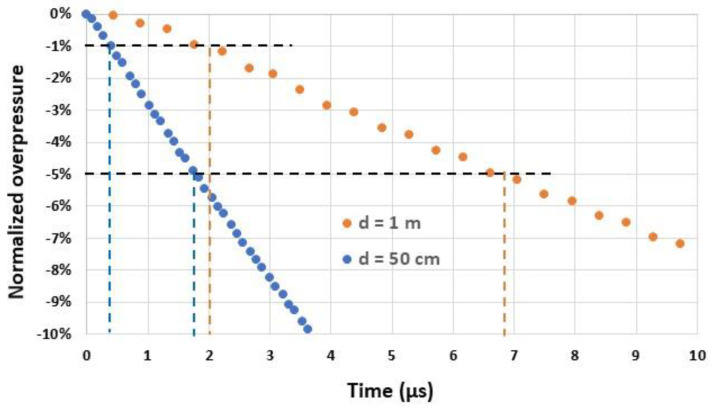
Normalized overpressure $\left[\left(P_{r}-P_{r-max}\right)/P_{r-max}\right]$ in the side-on configuration as a function of time t for two distances *d* (50 cm and 1 m) from an explosive charge of 1 kg of trinitrotoluene. At the origin t=0, the overpressure reaches its peak value $P_{r-max}$ ($P_{r-max}=33.5\;\mathrm{bar}$ at a distance of 50 cm and 8.7 bar at 1 m).

Simulations are performed with in-house CEA-DAM hydrodynamic codes to determine the time variation in the overpressure [24,25]. An example of such variation in free space is shown in Figure 3 for the explosion of a charge of trinitrotoluene of 1 kg and for charge-to-sensor distances d of 0.5 m and 1 m. From these data, we can derive the minimal response time required by the sensor to measure $P_{r-max}$ with the required accuracy. For d=50 cm (100 cm), an accuracy of 1% and 5% needs sensors with a response time of 0.3 µs (2 µs) and 1.7 µs (6.8 µs), respectively (see Table 2). We can conclude that sensors with a response time lower than 1 µs are needed for several configurations. To date, very short response times in side-on configurations have not yet been achieved by commercial sensors.

The time-domain response $K_{DYN}\left(t\right)$ of a pressure sensor subjected to a pressure step is generally modelled by a normalized transfer function of second order, as follows:

(1) $$K_{DYN}\left(t\right)=1-\frac{\exp\left(-2\pi \;f_{0}\;\xi \;t\right)}{\sqrt{1-\xi^{2}}}\sin\left(2\pi \;f_{0}\;\sqrt{1-\xi^{2}}\;t+arccos\;\xi \right)$$

where $f_{0}$ is the resonant frequency and ξ (<1) is the damping factor. The response $K_{DYN}$ is normalized, such that $K_{DYN}\left(t\to \infty \right)=1$.

For ξ < 0.2, Equation (1) can be approximated by the following equation:

(2) $$K_{DYN}\left(t\right)≅1-\exp\left(-\frac{\pi f_{0}}{Q}t\right)\sin\left(2\pi \;f_{0}\;t+\arccos\frac{1}{2Q}\right)$$

where Q=1/(2ξ) denotes the quality factor.

**Figure 4:**
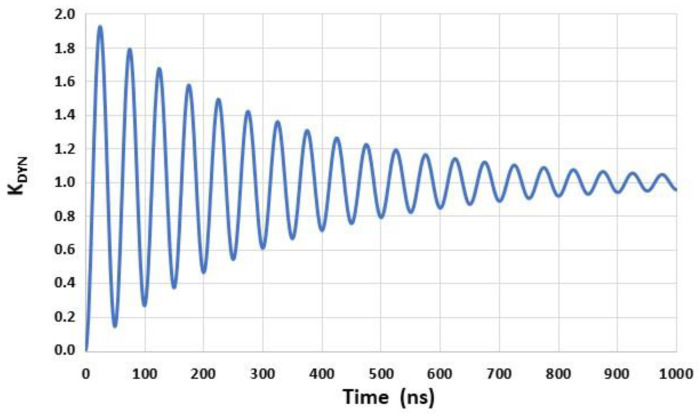
Example of a simulated second-order time-domain response ($K_{DYN}\left(t\right)$) of a sensor subjected to a pressure step for $f_{0}=20\;\mathrm{MHz}$ and Q=20 (ξ=0.025).

An example of the response $K_{DYN}\left(t\right)$ derived from Equation (2) is shown in Figure 4 for $f_{0}=20\;\mathrm{MHz}$ and Q=20 (ξ=0.025).

The response time at *x*% $\left(t_{r-x\%}\right),$ during which the response $K_{DYN}\left(t\right)$ lies between (1 − *x*%) and (1 + *x*%), may be estimated from Equation (2), as follows:

(3) $$t_{r-x\%}≅1.47\frac{Q}{f_{0}}\left(1-{log}_{10}\sqrt{x}\right)$$

**Figure 5:**
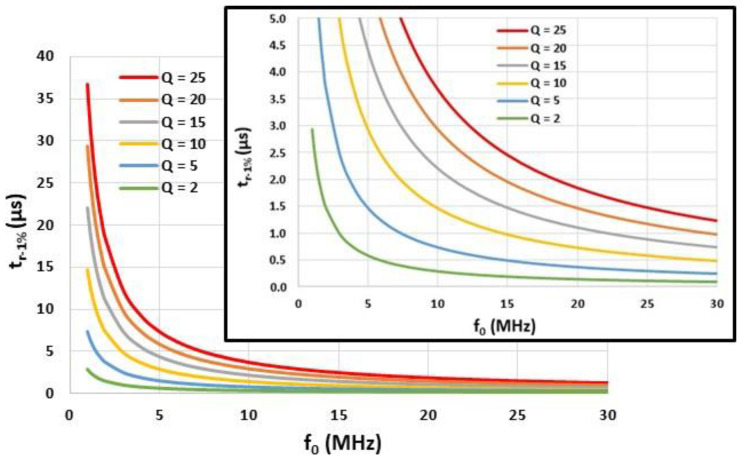
Response time $t_{r-1\%}$ computed from Equation (3) as a function of the resonant frequency $f_{0}$ and for different quality factors Q.

Figure 5 displays the response time $t_{r-1\%}$, computed from Equation (3), as a function of the resonant frequency $f_{0}$ and for various quality factors Q. If $t_{r-1\%}$ is required to be smaller than 1 µs, then the resonant frequency $f_{0}$ must be greater than 1.5 Q and specifically, this frequency must be greater than 15 MHz for Q > 10. If $t_{r-5\%}$ (instead of $t_{r-1\%}$) is required to be smaller than 1 µs, $f_{0}$ can be lower (10 MHz instead of 15 MHz).

In the previous analysis, the pressure generated by the blast wave is simultaneously applied on the entire membrane surface of the sensor. This is the case in practice for face-on configurations. However, for side-on configurations, which are usually used for measurements in free space, the dimension $l_{P}$ of the membrane (in the direction of blast wave propagation) plays a crucial role in the dynamic response of the sensor. The time $t_{P}$ required by the blast wave to travel the distance $l_{P}$ is inversely proportional to the velocity of the shock wave $V_{SW}$. This velocity is approximately given by the following equation [2]:

(4) $$V_{SW}=C_{sound}\sqrt{1+\frac{\gamma +1}{2\gamma }\;\frac{P_{r-max}}{P_{atm}}}$$

where *C_sound_* is the sound velocity in the unshocked medium (*C_sound_* = 343.4 m/s for the absolute pressure of 1 bar and temperature of 20 °C) and *γ* is the polytropic coefficient (*γ* = 1.402 in air).

**Figure 6:**
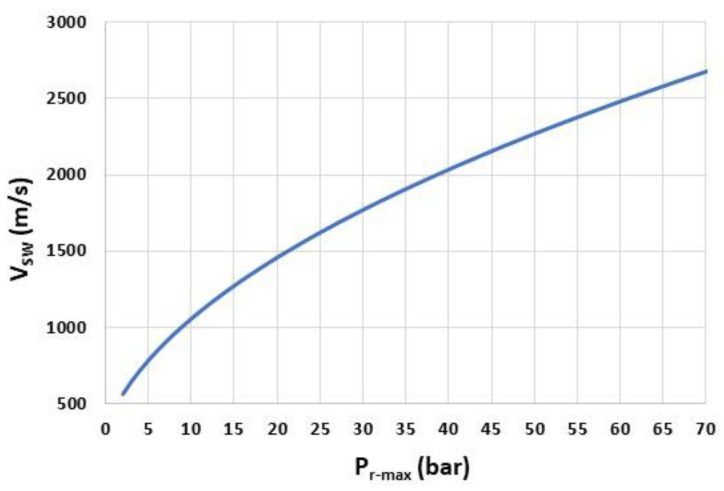
Shock-wave velocity $V_{SW}$ as a function of the overpressure peak $P_{r-max}$.

**Figure 7:**
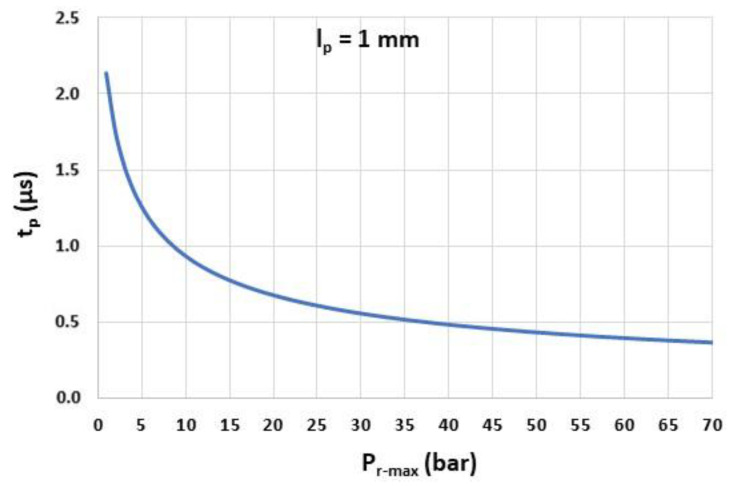
Travel time $t_{P}$ as a function of the overpressure peak $P_{r-max}$ ($l_{P}=1\;\mathrm{mm}$).

Figure 6 displays the velocity of the shock wave $V_{SW}$, computed from Equation (4), for $P_{r-max}$ between 1 bar and 70 bar and for $l_{P}=1\;\mathrm{mm}$. In these pressure ranges, the travel time $t_{P}$ is between 0.4 µs ($P_{r-max}=70\;\mathrm{bar}$) and 2.1 µs ($P_{r-max}=1\;\mathrm{bar}$) (Figure 7). In order to avoid a decrease in the response time of the sensor, especially for low $P_{r-max}$, $l_{P}$ must, therefore, be well below 1 mm.

## 3. Measurement Setup for the Characterization of Pressure Sensors in a Shock-Wave Tube

**Figure 8:**
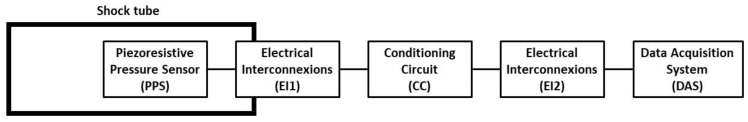
Diagram of the measurement setup used for the shock-wave tube characterization of a pressure sensor.

The measurement setup used to characterize the pressure sensors in the face-on configuration consists of the following three main blocks (Figure 8):

- The pressure transducer composed of a thin silicon membrane and piezoresistive strain gauges (output impedance of a few kilo-Ohms);
- The conditioning electronic circuit for impedance matching purposes and for providing a sufficient gain–bandwidth product. This circuit also guarantees the transducer power supply.
- The acquisition system used to sample the measurement signal. The input impedance of this system is 50 Ω.

The conditioning circuit was implemented on a separate electronic board and placed outside the shock-wave tube. The electrical connections EI1 between the multiple outputs of the transducer and inputs of the conditioning circuit may degrade the dynamic response of the measurement set up, due to impedance mismatches. From a preliminary study (not shown here), we reached a good trade-off between the gain and the frequency bandwidth of the conditioning circuit from setting the input impedance of the circuit to approximately 300 Ω. In this configuration, the capacitances of the electrical interconnections EI1 can then be neglected and the interconnections can be modeled by series inductances. Moreover, a shielded RG58 cable (EI2) was used to connect the outputs of the conditioning circuit and inputs of the acquisition system (EI2 interconnections will be neglected in the following section as the impedances are equal to 50 Ω over the entire line).

## 4. Piezoresistive Pressure Sensor

### 4.1. Description of the Sensor

**Figure 9:**
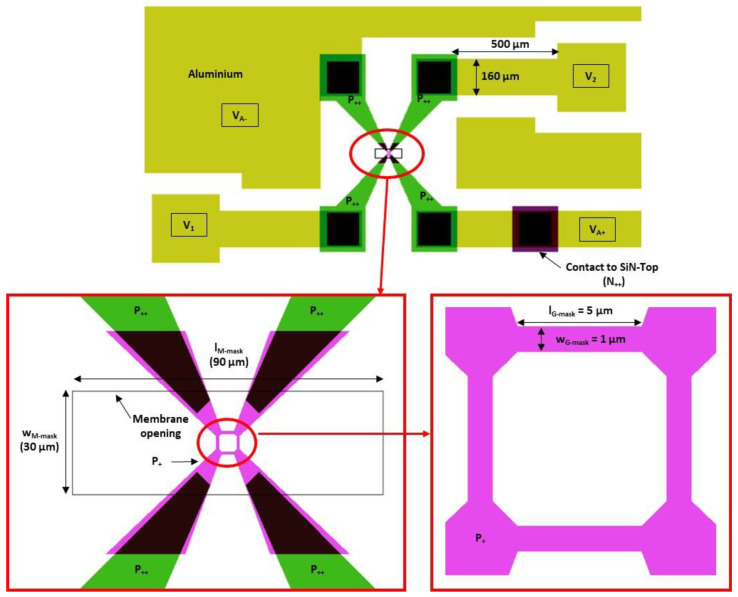
Top view of the mask used in the technological process.

**Figure 10:**
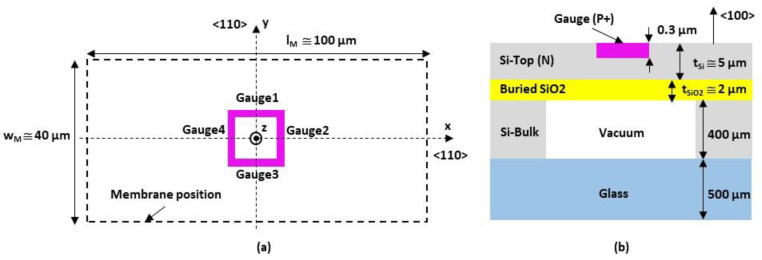
(**a**) Top view of the Wheatstone bridge reported at the center of the membrane surface; (**b**) cross-sectional view of the transducer.

The silicon pressure transducer is shown in Figure 9 and Figure 10. It is composed of the following three main parts:

- The monocrystalline N-type 5 µm thick silicon membrane. The mask opening values for the membrane fabrication are as follows: $w_{M-mask}=30\;µm$ by $l_{M-mask}$ = 90 µm;
- Four monocrystalline P-type silicon gauges implanted into the N-type silicon membrane and located at its center with a Wheatstone bridge configuration. The mask opening values for the gauge fabrication are as follows: $w_{G-mask}=1\;µm$ by $l_{G-mask}=5\;µm$. The isolation of the gauges between them is obtained from reverse polarization of the P/N junction ($V_{A+}-V_{A-}>0)$;
- The reference cavity with a vacuum.

### 4.2. Technological Process

The pressure transducers were fabricated on silicon-on-insulator (SOI) wafers, whose characteristics are given in Table 3.

**Figure 11:**
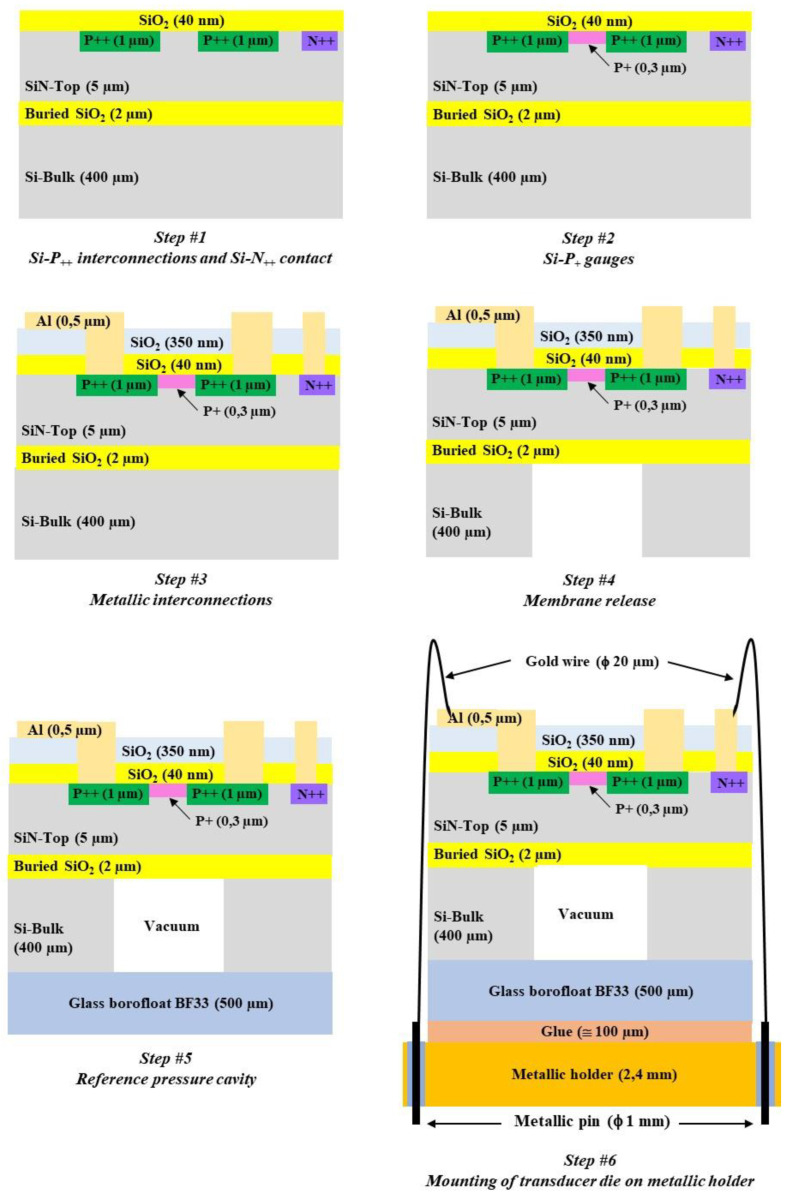
Main steps of the technological flow chart for sensor fabrication.

The main steps of the fabrication process are sketched in Figure 11. SOI wafers are first cleaned with a piranha solution (98% H_2_SO_4_/30% H_2_O_2_ 1:1 *v*/*v*) to remove the possible presence of organic contaminants, and next with hydrofluoric acid (HF) to remove the oxide layer created by the piranha solution.

Step #1 is dedicated to the implementation of the low-resistivity electrical interconnections (P_++_) with boron implantation and to the low-resistivity electrical contact on SiN-Top (N_++_) with phosphorus implantations. The two implantations are performed with the same parameters (energy = 50 keV; dose = 10^16^ at/cm^2^). A 40 nm thick thermal silicon dioxide (SiO_2_) layer is created before the implantations in order to avoid exodiffusion of the dopants. Annealing at 1000 °C is then performed for one hour in order to stimulate the electrical activation of the dopants. The junction depth is 1 µm.

Step #2 is focused on the fabrication of the piezoresistive gauges (P_+_) with boron implantation (energy = 20 keV; dose = 5 × 10^14^ at/cm^2^). Rapid activation annealing of the dopants is achieved at 1000 °C for 1 min, in order to keep the dopant close to the membrane surface where the stress is maximal during membrane deflection. The surface concentration is 3.5 × 10^19^ at/cm^3^, and the junction depth is 0.3 µm. These parameters provide a good trade-off between the gauge sensitivities to strain and to temperature. Due to photoresist lateral under-etching and lateral boron diffusion during annealing, the estimated gauges’ lateral dimensions are $w_{G}=1.5\;µm$ by $l_{G}=4.5\;µm$ [24].

Step #3 is dedicated to the implementation of the metallic interconnections. A 350 nm thick SiO_2_ layer is first deposited on the top side at 300 °C by plasma enhanced chemical vapor deposition (PECVD) in order to ensure good electrical insulation between silicon and future metallic interconnections. After the opening of the two SiO_2_ layers by buffered hydrofluorydric solution up to the silicon surface, a 500 nm thick aluminum layer is deposited by thermal evaporation. The annealing of the metal is then performed for 30 min at 450 °C, in order to achieve good adhesion and to provide a low contact resistance.

Step #4 concerns the silicon membrane fabrication. The 400 µm thick Si-Bulk is etched from the back-side up to the buried-SiO_2_ etch-stop layer by deep reactive ionic etching (DRIE) equipment. The average Si etch-rate is 4.5 µm/min with a selectivity ratio with SiO_2_ around 70. Due to the technological process, the lateral membrane dimensions are increased by approximatively 10 µm [23].

Step #5 is dedicated to the realization of the vacuum reference pressure cavity under the silicon membrane. Anodic bonding (temperature = 350 °C; voltage = 600 V) is performed with a 500 µm thick Borofloat-B33 glass substrate on the back-side of the SOI substrate using an AML (A-INTE) wafer bonder.

Step #6 consists of packaging the transducer die using a 2.4 mm thick stainless steel TO3 holder with an Ni-Fe pin (length = 13.1 mm; diameter = 1 mm). After dicing, the silicon/glass die is glued with a thermosetting epoxy (Epotek H70E) on the holder, followed by annealing at 80 °C for 90 min. Wire bonding is then performed between the transducer pads and holder pins using 20 µm diameter gold wires.

**Figure 12:**
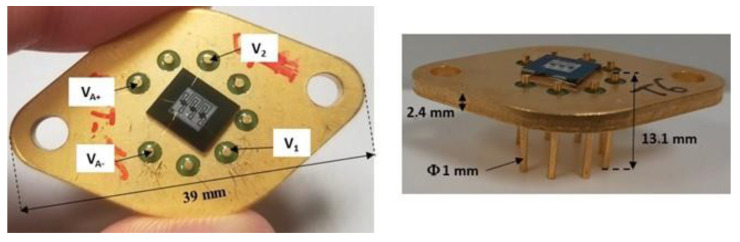
Photographs of the transducer die on its TO3 stainless steel holder.

A photograph of the fabricated pressure sensor is shown in Figure 12.

### 4.3. Electrical Model and Simulation Results

**Figure 13:**
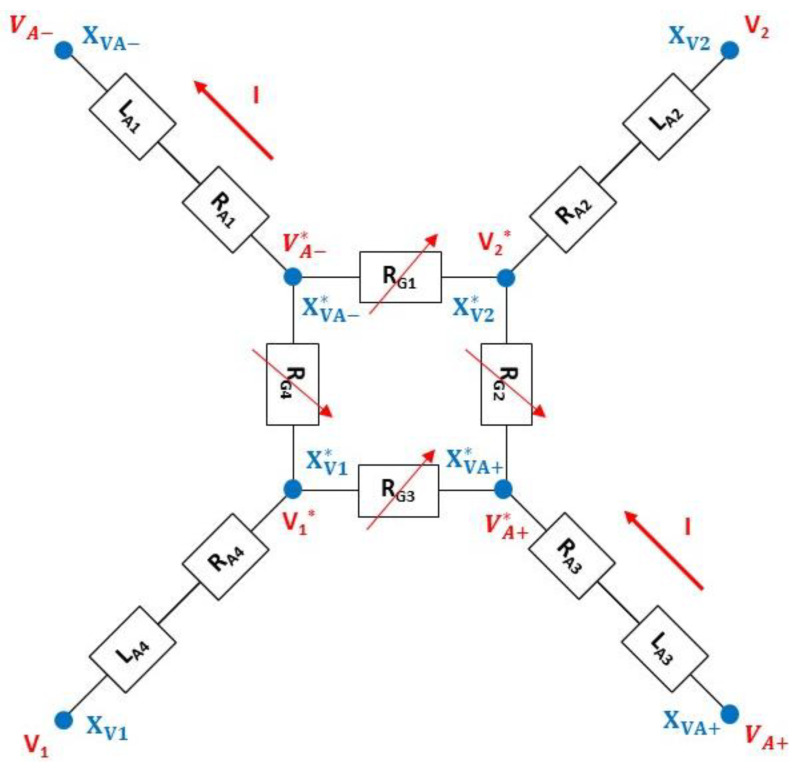
Electrical model of the pressure sensor.

The electrical model of the pressure transducer is shown in Figure 13 and includes the following two main parts:

- The four variable gauge resistors ($R_{G1},\;R_{G2,\;}\;R_{G3},\;R_{G4})$, which correspond to the resistors of the Wheatstone bridge. The four resistors are assumed to be identical for a zero differential pressure between the two sides of the diaphragm $\left(R_{G10}=R_{G20}=R_{G30}=R_{G40}\right)$;
- The four electrical access resistors in the Wheatstone bridge, which consist of the resistor $R_{A}$ in series with the inductance $L_{A}$. The values of these impedances are assumed to be independent of pressure.

**Figure 14:**
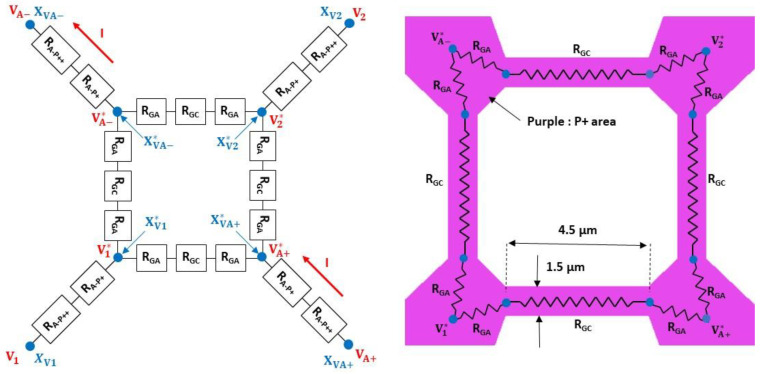
Electrical model for constitutive resistor without differential pressure applied on the membrane.

The electrical model of the resistors, without differential pressure applied on the membrane, is shown in Figure 14. Each branch within the Wheatstone bridge consists of the following two resistors: the $R_{GC}$ resistor associated with the 1.5 µm × 4.5 µm gauge and the $R_{GA}$ resistors used to connect $R_{GC}$ to the electrical closure point of the Wheatstone bridge. The access resistors in the Wheatstone bridge are considered to be identical in the four branches ($R_{A1}=R_{A2}=R_{A3}=R_{A4}=R_{A}$) and consist of the following two resistors in series: the $R_{A-P++}$ resistor associated with the highly doped silicon accesses (*P*_++_) and the $R_{A-P+}$ resistor, which originates from the silicon areas with gauge doping (see Figure 9).

**Figure 15:**
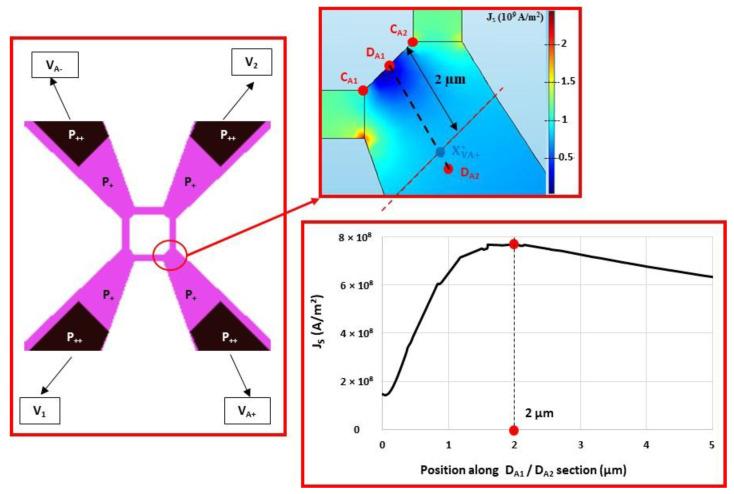
Distribution of surface current density $J_{S}$ around $X_{VA+}^{}$ point.

Electrical simulations were carried out using COMSOL Multiphysics software to determine the values of the various resistances. The first step is to determine the position of the $X_{VA-}^{*}$ and $X_{VA+}^{*}$ points inside the *P*_+_ interconnections. For this purpose, the transducer (see Figure 9) is simulated and the surface current density $J_{S}$ is extracted in the $\left[X_{VA+}^{*}-X_{VA+}\right]$ segment (see Figure 15). An inflection point on $J_{S}$ is found along the $\left[D_{A1}^{}-D_{A2}^{}\right]$ segment located at about 2 µm from $D_{A1}^{}$. This inflection point can be attributed to the concentration of the current lines up to the electric point of closure of the Wheatstone bridge, followed by a decrease due to a larger interconnection. As a first approximation, we consider that the electrical bridge closure zone is a line parallel to the $\left[C_{A1}^{}-C_{A2}^{}\right]$ segment and is located at a distance of 2 µm from $D_{A1}^{}$. Given the symmetry of the transducer, the same approach can be applied in the $\left[X_{VA-}^{*}-X_{VA-}^{}\right]$ branch. As there is no current flowing through the $\left[X_{V1}^{*}-X_{V1}^{}\right]$ and $\left[X_{V2}^{*}-X_{V2}^{}\right]$ branches, the bridge closure area in these branches will be considered to be similar to that shown in Figure 15.

**Figure 16:**
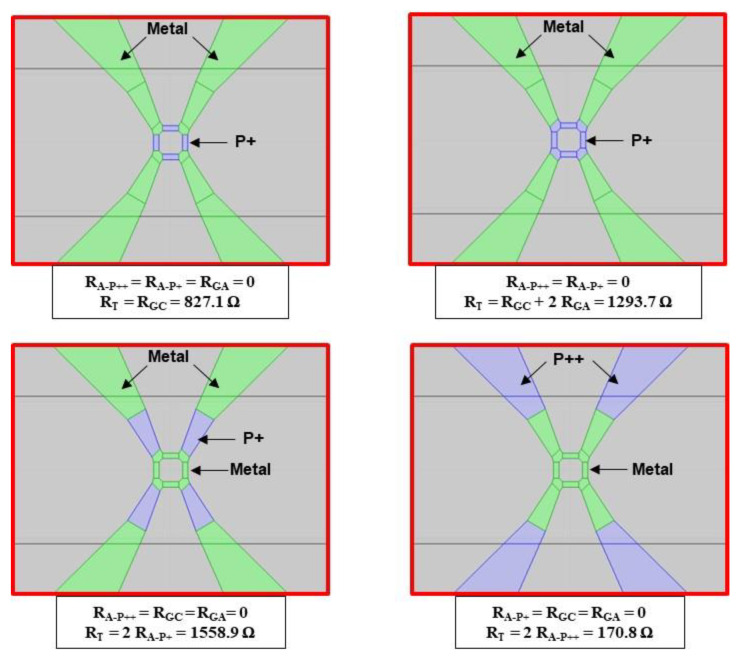
Four cases simulated using COMSOL software to determine the resistances (green: Metal; blue: *P*_+_ or *P*_++_).

Four configurations are simulated by replacing different parts of silicon with metal, and the $R_{T}^{}$ resistance between $X_{VA+}^{}$ and $X_{VA-}^{}$ is then determined (Figure 16). The simulation results are given in Table 4. The total resistance of the transducer $R_{T0}^{}$ $(R_{T0}^{}=R_{G0}^{}+2\;R_{A}^{}$) is 3023 Ω, and is very close to the measured value (3200 Ω). The access resistance $R_{A}^{}$, mainly composed of the silicon area with the doping of the gauges, is 865 Ω. This value is not negligible compared to the resistance $R_{G0}^{}$ (1294 Ω) of the gauges. The pressure sensitivity $K_{S}^{}$ of the sensor is, therefore, about twice as low as the pressure sensitivity of the gauges $K_{G}^{}$ ($K_{S}^{}=$0.43 $K_{G}^{}$).

The variation in the gauge resistances with the static differential pressure $P_{S}$ (applied between the two sides of the membrane) is given by Equations (5) and (6). As the gauges are located at the center of the membrane, the $K_{G}^{}$ factor is considered to be identical for all four gauges, and consequently

(5) $$R_{G1}=R_{G3}=R_{G0}\left(1+K_{G}*P_{S}\right)$$

(6) $$R_{G2}=R_{G4}=R_{G0}\left(1-K_{G}*P_{S}\right)$$

The dynamic sensitivity $K_{G-DYN}$ of the gauges to a pressure step is then given by the following equation:

(7) $$K_{G-DYN}=K_{G}*\;K_{DYN}$$

where $K_{DYN}$ is the transfer function of a two-order low-pass filter and models the mechanical behaviour of the membrane given by Equation (2).

**Figure 17:**
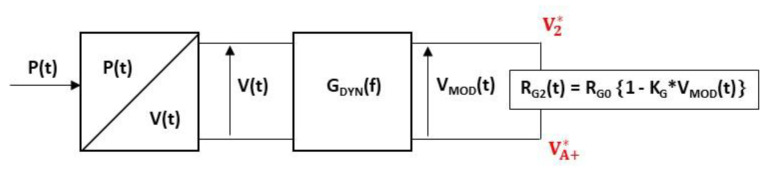
Electrical model of variable resistance implemented in ADS software (example for *R_G_*_2_ gauge).

The dynamic sensitivity model of the gauge is implemented on the ADS simulator (see Figure 17). The time-varying pressure P(t) applied to the membrane is converted into a variable voltage V(t) for simulation purposes, with an arbitrary conversion factor of 1 bar/V. The instantaneous value of gauge resistance $R_{G}\left(t\right)$ is a function of the modulation voltage $V_{MOD}\left(t\right)$, resulting from the low-pass transfer function $G_{DYN}\left(f\right)$, given by Equation (8), and voltage V(t).

(8) $$G_{DYN}=\frac{1}{1+\left(j\;\frac{1}{Q}\;\frac{f}{f_{0}}\right)+{\left(j\;\frac{f}{f_{0}}\right)}^{2}}$$

where *f* denotes the frequency and $j^{2}=-1$.

On each access branch of the Wheatstone bridge, the input inductances consist of the following four inductances in series: $L_{A-SI}$, $L_{A-AT}$, $L_{A-GW}$ and $L_{A-NiFe}$, which model doped silicon interconnections, the aluminum metal tracks, the gold micro-wires, and the nickel–iron alloy pins of the TO3 holder, respectively.

The different inductances are estimated from Equation (9) for surface conductors [26] and Equation (10) for cylindrical conductors [27].

(9) $$L_{A}=\frac{\mu_{o}}{2\;\pi }\;L_{g}\;\left[\ln\left(\frac{2\;l_{g}}{w+t}\right)+0.5\right]$$

(10) $$L_{A}=\frac{\mu_{o}}{2\;\pi }\;l_{g}\;\left[\ln\left(\frac{2\;L_{g}}{Ø}\left(1+\sqrt{1+{\left(\frac{Ø}{2\;L_{g}}\right)}^{2}}\right)\right)-\sqrt{1+{\left(\frac{Ø}{2\;L_{g}}\right)}^{2}}+\frac{\mu_{R}}{4}+\frac{Ø}{2\;L_{g}}\right]$$

where $l_{g},\;w,\;t$ and Ø denote the length, the width, the thickness and the diameter of the conductor, respectively, and $\mu_{0}$ and $\mu_{R}$ denote the magnetic permeability of the vacuum and the relative magnetic permeability of the conductor ($\mu_{R}$ ≅ 1 for non-magnetic metals), respectively.

The dimensions associated with the inductances are assumed to be identical for the four access branches of the Wheatstone bridge and are calculated for the longest access lengths (Table 5). The equivalent inductance $L_{A-PPS}$ of each branch is 15 nH and is mainly related to the TO3 pins.

## 5. Electrical Interconnections between Sensor and Conditioning Circuit

**Figure 18:**
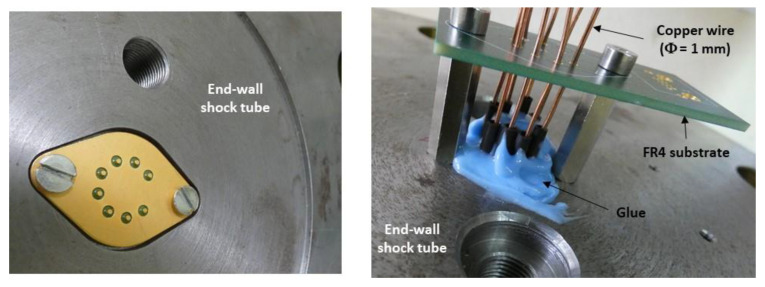
View of the TO3 holder embedded inside the end-wall shock-wave tube (**left**) and the electrical interconnection outside the end-wall shock-wave tube (**right**).

**Figure 19:**
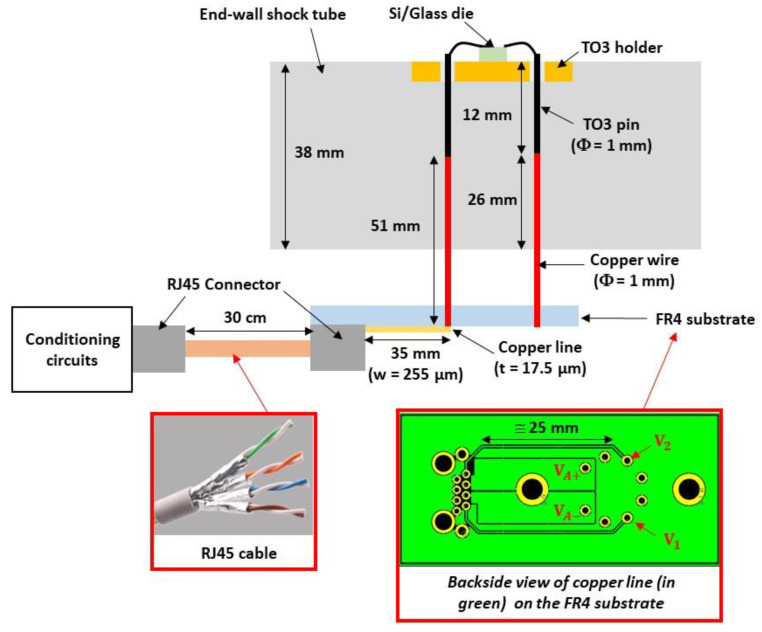
Description of electrical interconnections between the transducer and the conditioning circuit.

The electronic board that contains the conditioning circuits is located outside the shock-wave tube. Figure 18 shows a view of the sensor with the electrical interconnections passing through the end-wall of the shock-wave tube. The electrical interconnections between these circuits and the sensor include the following three main parts (Figure 19):

- The 1 mm diameter copper wires, soldered to the pins of the TO3 holder. These wires cross the wall of the shock-wave tube;
- The copper tracks deposited on a FR4 holder card; one end is soldered to the 1 mm diameter copper wires and the other end is connected to an RJ45 connector;
- The shielded RJ45 cable that contains four pairs of twisted copper wires of 287 µm diameter. Each pair is shielded by an aluminum foil to minimize the electromagnetic coupling between the different paths. One pair is used for the Wheatstone bridge power supply and the other pair for the sensor output. The end of the cable is connected to an RJ45 connector. Each twisted pair has a characteristic impedance of 100 Ω. This type of cable has a frequency bandwidth greater than 250 MHz.

The values of the different inductances, calculated using Equations (9) and (10), are reported in Table 6. For the RJ45 cable with twisted copper wires, the inductance is given by the technical datasheet (525 nH/m). The total inductance $L_{A-EI1}$ of the interconnections between the sensor and the conditioning circuit is 247 nH. The electrical resistances of these metallic interconnections are negligible.

## 6. Conditioning Circuit

### 6.1. Description of Conditioning Electronic Circuit and Simulation Results

The conditioning circuit used an operational amplifier (OA) with differential outputs (THS4520RGTT from Texas Instruments). This differential option allows the signal to be isolated from ambient electrical noise. It will also be possible to subsequently use an offset correction device followed by a differential amplifier stage. The main characteristics of the OA are shown in Table 7. The OA has a gain–bandwidth product of 620 MHz and a low-frequency common mode rejection ratio of 84 dB. The OA is designed to acquire voltage signals up to 5 V within a dynamic range of 570 mV/ns and has very low voltage noise (2 nV·Hz^−0.5^).

**Figure 20:**
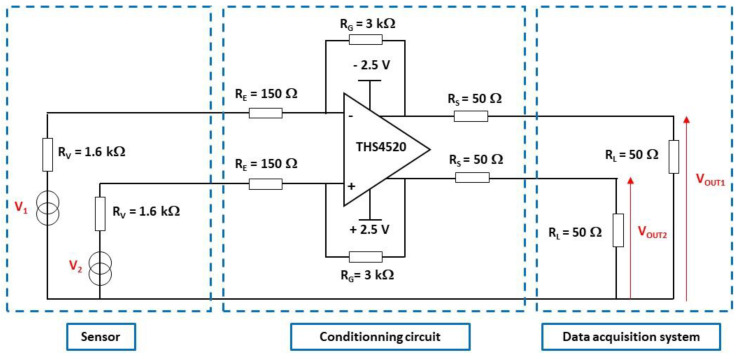
Architecture of the conditioning circuit embedded inside the measurement setup.

The architecture of the conditioning circuit is shown in Figure 20 with the sensor and the acquisition system (interconnections EI1 and EI2 are not shown). The sensor is modelled, for each channel, by a resistor $R_{V}$ (its value is half the measured resistance $R_{T0}$ of the transducer, see Table 4). The acquisition system is modelled, for each channel, by a load resistance $R_{L}$ of 50 Ω.

The two channels of the OA are connected as an amplifier/inverter. The ratio between $R_{G}$ and $R_{E}$ determines the open-circuit voltage gain $G_{0}$ of the circuit. This gain is set to 20 V/V to ensure high gain, while preserving the frequency bandwidth above the resonant frequency (<30 MHz) of the transducer. The input impedance $R_{E}$ of 150 Ω is found to be a good trade-off between the high sensitivity of the pressure transducer (including the conditioning circuit) and the large bandwidth of the measurement setup. The output impedance $R_{S}$ is set to 50 Ω for impedance matching purposes.

The low-frequency gain of the differential mode $G_{DM-LF}$ of the conditioning circuit is given by Equation (11). The calculated gain is 0.86 (−1.3 dB). The various $G_{DM-LF}$ factors are reported in Table 8.

(11) $$G_{DM-LF}=\frac{V_{OUT2}-V_{OUT1}}{V_{1}-V_{2}}=\frac{1}{1+\frac{R_{V}}{R_{E}}}\;\frac{R_{G}}{R_{E}}\;\frac{R_{L}}{R_{L}+R_{S}}=G_{IN}\;G_{0}\;G_{OUT}$$

### 6.2. Experimental Results

**Figure 21:**
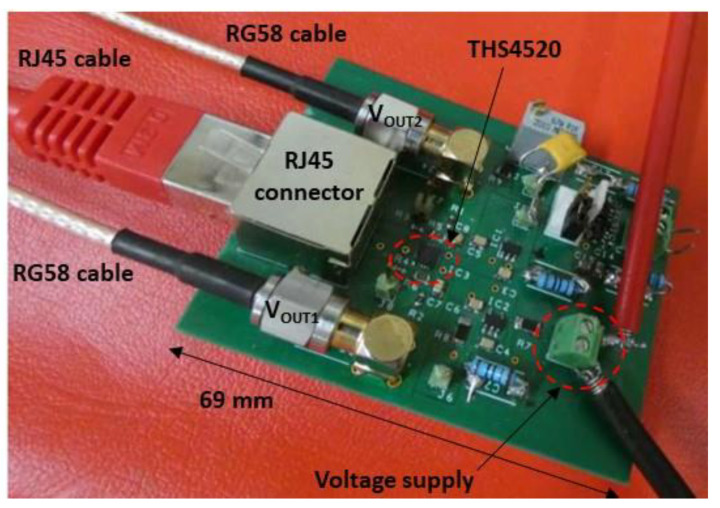
View of the printed circuit board of the conditioning circuit.

The conditioning circuit was manufactured using discrete components mounted on a printed circuit board (PCB) (Figure 21). In order to minimize impedance mismatches on the two measurement channels, the PCB layout was carefully designed and the implemented impedances were chosen to be precisely matched. Indeed, these mismatches can strongly decrease the common mode rejection ratio of the circuit.

**Figure 22:**
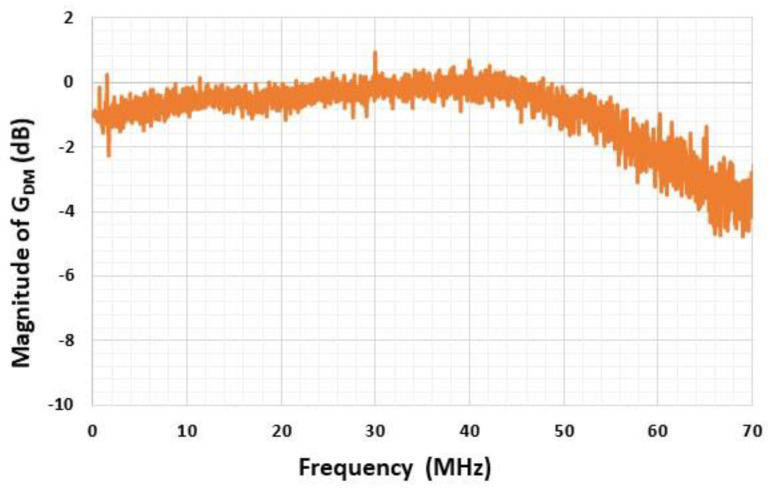
Measured magnitude of the differential gain of the conditioning circuit versus frequency.

The differential mode gain $G_{DM}$ of the conditioning circuit is characterized under the impedance load conditions (input and output), as defined in Figure 20, for frequency up to 70 MHz (Figure 22). The pulse differential voltage of 1 V (±0.5 V) is applied between the two input channels of the circuit for 60 ns. The input signal rise time is 5 ns. The differential output signal of the circuit is measured with a sampling rate of 2.5 GS/s and the results are averaged for 100 identical measurements. The magnitude of the measured low-frequency gain $G_{DM-LF}$ is 0.89 (−1 dB), and is in good agreement with the calculated gain. The cut-off frequency (at −3 dB) of the transfer function is 65 MHz, which is much higher than the resonant frequency of the sensor membrane (<30 MHz).

## 7. Results and Discussion

**Figure 23:**
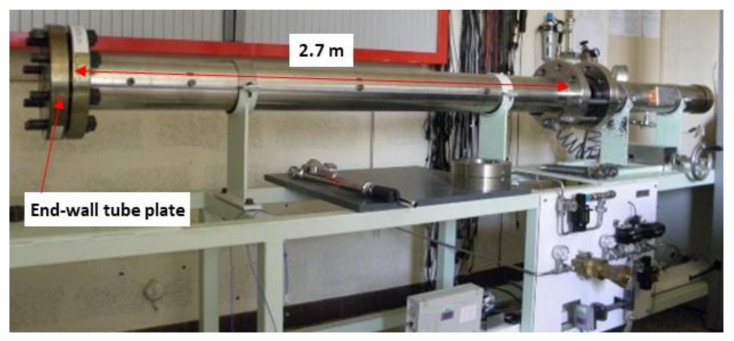
Photograph of the shock-wave tube used at CEA-Gramat.

The shock-wave tube used for the dynamic characterization of pressure sensors is shown in Figure 23. The length of the driven section is 2.70 m and the internal diameter of the tube is 11 cm.

**Figure 24:**
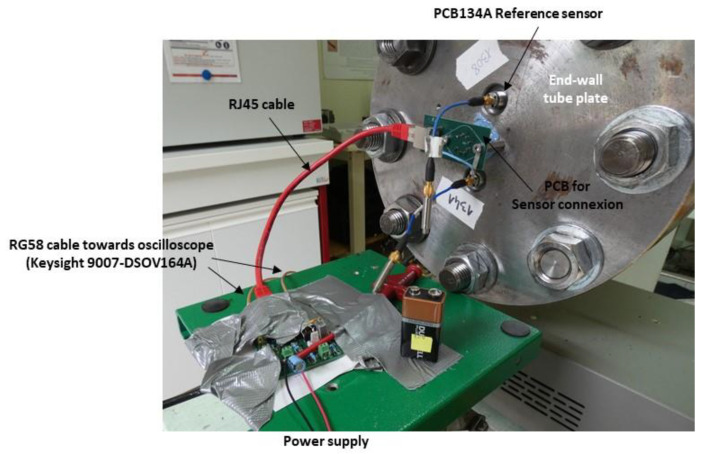
Experimental setup for dynamic characterization of pressure sensors.

**Figure 25:**
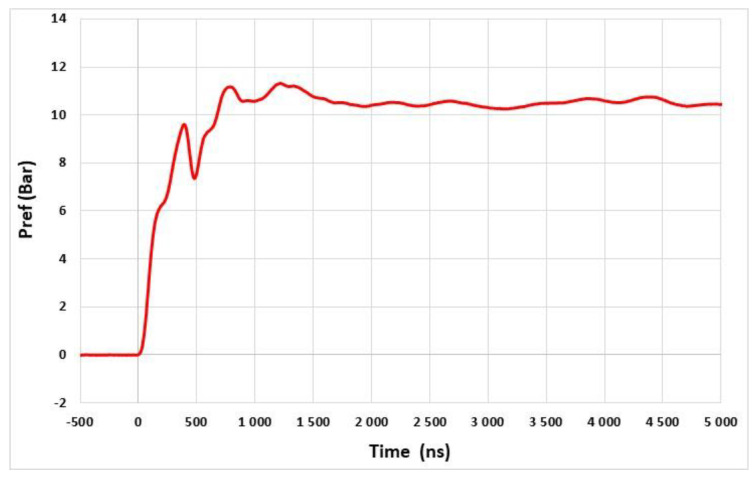
Response of the reference pressure sensor PCB134A.

The setup used for the dynamic characterization of the system is shown in Figure 24. The relative pressure $P_{r}\;\left(P_{r}=P-P_{atm}\right)$ is measured using a reference sensor (PCB Piezotronics 134A24) located close to the sensor under test. The pressure step $P_{r}$ applied is 10 bars (Figure 25). The differential output voltage of the system $V_{OUT}$ is measured using an oscilloscope (Keysight 9007 DSOV164A) with a differential input impedance of 100 Ω. The sampling rate is set to 2 GS/s (time step of 0.5 ns).

**Figure 26:**
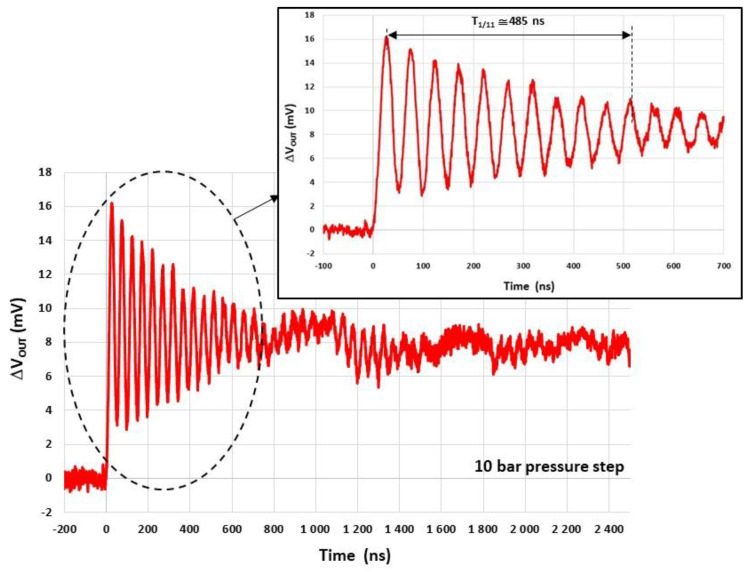
Measured system response $\Delta V_{OUT}$ to a pressure step of 10 bars.

Figure 26 shows the time variation in the measured response $\Delta V_{OUT}$ of the system for the pressure step $P_{r}$ of 10 bars. A damped oscillatory response is observed up to 700 ns after the arrival of the shock wave at the sensor. Thereafter, drifts appear, which may be due to the deformation of the TO3 metallic holder [23]. The resonant frequency $f_{0}=10/t_{1-11}≅20.6\;\mathrm{MHz}$ is estimated from the duration $t_{1-11}$ between the first and the eleventh maximum value of the oscillatory response. This is the highest resonant frequency reported in the literature. The steady state value $\Delta V_{OUT-SS}≅$ 8.4 mV is estimated by averaging the signal value from 0 ns to 700 ns.

**Figure 27:**
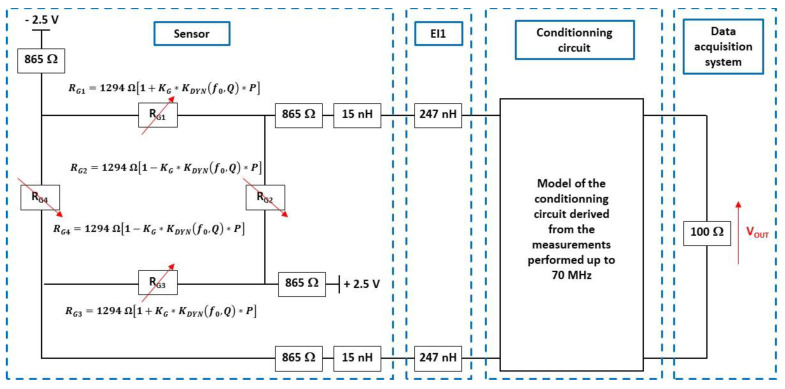
Electrical model of entire measurement setup implemented in ADS software.

**Figure 28:**
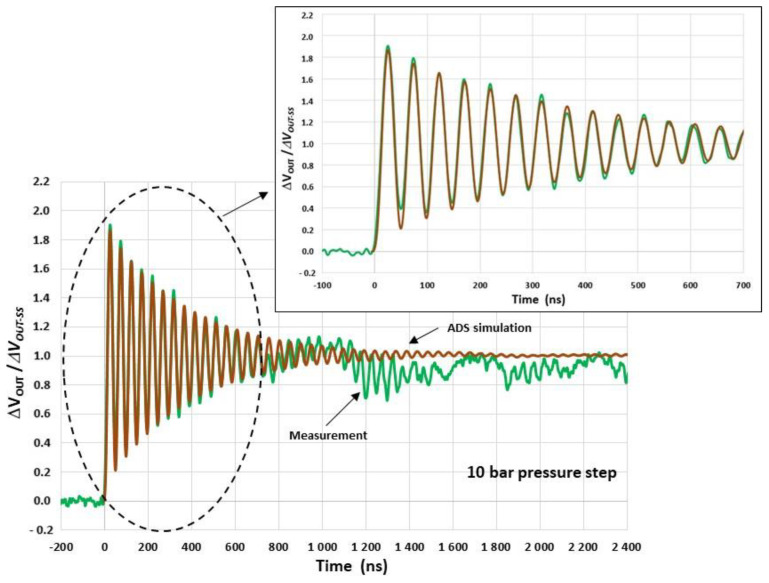
Simulated (ADS) and measured (after high-frequency filtering) responses of the system. ($K_{G}\;$= 435 µV/V/bar, $f_{0}$ = 20.6 MHz, Q = 24 and $\Delta V_{OUT-SS}$ = 8.4 mV).

In order to filter the high-frequency noise in the signal response, numerical elliptic second-order low-pass filtering with a cut-off frequency of 30 MHz is applied. The resulting signal is then compared with the ADS simulation results of the entire measurement setup, whose characteristics are shown in Figure 27. For these simulations, $f_{0}$ and $\Delta V_{OUT-SS}$ are set to 20.6 MHz and 8.4 mV, respectively, and the conditioning circuit is modelled as detailed in Section 6.2. The parameters $K_{G}$ and Q ($K_{G}\;$= 435 × 10^−6^/bar, Q = 24) are derived from the interpolation of the measurement results. The simulated and measured responses are in good agreement up to 700 ns after the arrival of the shock wave at the sensor (Figure 28). Therefore, the conditioning circuit and the electrical interconnections do not significantly alter the damped oscillatory response of the membrane. Consequently, after removing undesirable drifts that appear 700 ns after the time arrival of the shock wave, we may expect a response time $t_{r-5\%}$ of 1.2 µs. This response time of 1.2 µs is found to be in agreement with the time (1.4 µs) obtained from the reference commercial sensor. However, the response time of commercial sensors in the incident mode is expected to be significantly longer due to the large size of the sensing element (see Section 2).

## 8. Conclusions

A piezoresistive pressure sensor with a 40 µm × 100 µm × 5 µm thick silicon rectangular membrane and 1.5 µm × 4.5 µm × 0.3 µm thick silicon gauges located on the membrane center was fabricated and its dynamic response was characterized using a shock-wave tube experiment. The measurement setup (sensor, interconnections, and conditioning circuit) was characterized and modelled using ADS software (ADS-2019).

The transient response of the pressure sensor to a pressure step of 10 bars was measured for face-on configurations using a shock-wave tube. Up to 700 ns after the arrival of the shock wave at the sensor, we have shown that the measured response can be modelled by the damped oscillatory response of the membrane. This result shows that the conditioning circuit and the electrical interconnections do not significantly alter the damped oscillatory response of the membrane. The obtained mechanical resonant frequency of the sensor of 20.6 MHz is the highest reported in the literature and consequently, a response time (at 5%) of 1.2 µs can be expected.

The next step of this work will be focused on the reduction in the undesirable drifts that occur after 700 ns in the dynamic response of the sensor. These drifts are probably due to the deformation of the sensor holder and consequently, a new type of packaging is under development to overcome this issue.

## Figures and Tables

**Table 1:** Performances (from datasheets provided by manufacturers) of commercial sensors dedicated to air blast monitoring ($T_{max}$ is the maximal operating temperature, $f_{0}$ is the resonant frequency, $t_{ri}$ is the sensor rise time; ${Ø}_{SE}$ is the diameter of the sensing element. Face-on and side-on modes correspond to the cases where the blast wave direction is normal and tangential to the surface of the sensors, respectively. NA stands for “not available data”).

Manufacturer	Reference	Material	Sensing Mode	Pressure Range (Bar)	*T_max_* (°C)	*f*_0_ (kHz)	*t_ri_* (µs)	*Ø*_SE_ (mm)
PCB Piezotronics	113B	Quartz	Face-on	3.5/69	135	>500	<1	NA
	134A	Tourmaline	Face-on	69	49	>1500	<0.2	NA
	137B	Quartz	Side-on	3.5/69	135	>400	<4 ^1^	5.6
Kistler	601C	Quartz	Face-on	1.5/250	120	>200	<1.4	NA
	603B	Quartz	Face-on	0/200	200	>400	<1	NA
	6233A	Quartz	Side-on	1.7/70	125	>300	<6	NA
Müller Instruments	M60	PVDF	Face-on	400	65	3900 ^2^	<0.06	3
	M100	PVDF	Face-on	400	65	NA	<0.1	1

^1^ For shock-wave velocity of 660 m/s ($P_{r-max}$ ≅ 3 bar). ^2^ Data from shock tube measurements performed at CEA-Gramat (France).

**Table 2:** Minimal response time required by the sensor to measure $P_{r-max}$ with the required accuracy.

$\mathrm{Accuracy}\;\mathrm{of}\;\mathrm{Expected}\;P_{r-max}\;$	*d* = 50 cm	*d* = 1 m
**5%**	1.7 µs	6.8 µs
**1%**	0.4 µs	2 µs

**Table 3:** Silicon-on-insulator wafer characteristics provided by the supplier.

**SiN-Top**	Orientation	(100)
	Doping type	N
	Doping level	4.8 × 10^15^ to 1.6 × 10^15^ at/cm^3^
	Thickness	(5.0 ± 0.5) µm
**Buried SiO_2_**	Thickness	(2.0 ± 0.1) µm
**Si-Bulk**	Orientation	(100)
	Thickness	(400 ± 15) µm

**Table 4:** Values of the different resistances shown in Figure 13 and Figure 14 and those derived from COMSOL simulations.

*R*_*GC*_	*R*_*GA*_	*R*_*G*0_ (*R*_*GC*_ + 2 *R*_*GA*_)	*R*_*A-P*+_	*R*_*A-P*++_	*R*_*A*_ (*R*_*A-P*+_ + *R*_*A-P*++_)	*R*_*T*0_ (*R*_*G*0_ + 2 *R*_A_)	*R*_*T*0_ (Measured)
827.1 Ω	233.3 Ω	1293.7 Ω	779.5 Ω	85.4 Ω	864.9 Ω	3023.4 Ω	3200 Ω

**Table 5:** Dimensions and estimated values of the different equivalent inductances inside of the sensor.

	Silicon Interconnection	Aluminum Track	Gold Wire	NiFe TO3 Pin
**Length**	*l_g_* = 350 µm	*l_g_* = 500 µm	*l_g_* = 5 mm	*l_g_* = 13.1 mm
**Width**	*w* = 60 µm	*w* = 160 µm		
**Thickness/diameter**	*t* = 1 µm	*t* = 0.5 µm	*Ø* = 20 µm	*Ø* = 1 mm
**Inductance *L_A_***	*L_A-SI_* ≅ 0.2 nH	*L_A-AT_* ≅ 0.2 nH	*L_A-GW_* ≅ 6.2 nH	*L_A-NiFe_* ≅ 8.5 nH
**Total inductance**	*L_A-PPS_* ≅ 15 nH			

**Table 6:** Dimensions and estimated values of the different parasitic inductances inside the EI1 interconnections.

	Copper Wire	Copper Line	RJ45 Cable
**Length**	*l_g_* = 51 mm	*l_g_* = 35 mm	*l_g_* = 30 cm
**Width**		*w* = 255 µm	
**Thickness/diameter**	*Ø* = 1 mm	*t* = 17.5 µm	*Ø* = 287 µm
**Inductance *L_A_***	*L_A-CW_* = 46.7 nH	*L_A-CL_* = 42.3 nH	*L_A-RJ_*_45_ = 157.5 nH
**Total inductance**	*L_A-EI_*_1_ = 246.5 nH		

**Table 7:** Main characteristics of the operational amplifier THS4520RGTT.

Gain–Bandwidth Product	Low-Frequency Common Mode Rejection Ratio	Slew Rate	Input Voltage Noise (*f* > 10 kHz)
620 MHz	84 dB	570 mV/ns	2 nV·Hz^−0.5^

**Table 8:** Low-frequency gains of the conditioning circuit (from Equation (11)).

*G*_*IN*_	*G*_0_	*G*_*OUT*_	*G*_*DM-LF*_
0.086	20	0.5	0.86 (−1.3 dB)

## Data Availability

Not applicable.
